# Computational Analysis of Specific MicroRNA Biomarkers for Noninvasive Early Cancer Detection

**DOI:** 10.1155/2017/4680650

**Published:** 2017-03-05

**Authors:** Tianci Song, Yanchun Liang, Zhongbo Cao, Wei Du, Ying Li

**Affiliations:** ^1^College of Computer Science and Technology, Key Laboratory of Symbol Computation and Knowledge Engineering of the Ministry of Education, Jilin University, Changchun 130012, China; ^2^Zhuhai Laboratory of Key Laboratory of Symbolic Computation and Knowledge Engineering of Ministry of Education, Zhuhai College of Jilin University, Zhuhai 519041, China

## Abstract

Cancer is a complex disease residing in various tissues of human body, accompanied with many abnormalities and mutations in genomes, transcriptome, and epigenome. Early detection plays a crucial role in extending survival time of all major cancer types. Recent advances in microarray and sequencing techniques have given more support to identifying effective biomarkers for early detection of cancer. MicroRNAs (miRNAs) are more and more frequently used as candidates for biomarkers in cancer related studies due to their regulation of target gene expression. In this paper, the comparative analysis is used to discover miRNA expression patterns in cancer versus normal samples on early stage of eight prevalent cancer types. Our work focuses on the specific miRNAs biomarkers identification and function analysis. Several identified miRNA biomarkers in this paper are matched well with those reported in existing researches, and most of them could serve as potential candidate indicators for clinical early diagnosis applications.

## 1. Introduction

Cancer is a highly complex disease that contains many abnormalities and mutations in the genomes, transcriptome, and epigenome. These abnormalities play important roles in the cancer cell growth [1]. Cancer is one of the leading causes of death all over the world. According to the world cancer report of 2014, there are about 14 million new cases and more than 8 million deaths related to cancer in 2012. The number will continue to rise if there are no effective prediction and treatment of cancer. It is expected that the number of new cases will rise to 22 million over the next two decades [2].

Early detection of cancer is undoubtedly important. During the development of cancer, some genes or proteins, associated with genetic mutations, transcription, or epigenetic alterations, could be detected from the tissue of cancer or inflammation when being compared to the normal tissue. These genes or proteins could give a quantitative measurement to the severity of a cancer. There have been many genes or proteins as biomarkers used in the clinical detection of cancer including alpha-fetoprotein (AFP) for liver cancer [3], BRCA1 and BRCA2 for breast and ovarian cancer [4], prostate specific antigen (PSA) for prostate cancer [5], and epidermal growth factor receptor (EGFR) for non-small-cell lung carcinoma [6].

In recent years, more and more researches on microRNA (miRNA) biomarkers have been published. miRNAs are small noncoding RNA molecules that contain 21–24 nucleotides. They play important roles in the posttranscriptional regulation of target gene expression [7]. The miRNA biomarker identification has been extensively studied in recent years. High-throughput microarray and sequencing techniques are widely used for transcriptome analysis. We can acquire lots of transcriptome information for various kinds of cancers on gene expression level from public databases, such as Gene Expression Omnibus (GEO) [8], Stanford Microarray Database (SMD) [9], Oncomine [10], and the Cancer Genome Atlas (TCGA) [5]. Dysregulation of miRNA expression is important for cancer development through various mechanisms including deletions, amplifications, or epigenetic silencing [11]. The circulating miRNAs are suggested to be effective indicators of disease. They are important for clinical applications such as disease diagnostics, monitoring therapeutic effect, and predicting recurrence in cancer patients [12]. Circulating miRNAs are widely used as biomarkers for various human cancers, such as prostate cancer and breast cancer [13–16]. Some gene or miRNA biomarkers are identified using statistical methods to the high-throughput omics data. MiRNAs are widely used as biomarkers for human cancers. Resnick et al. acquired 21 differentially expressed miRNAs through comparing microarray data between 28 patients with ovarian cancer and 15 normal samples. Five differentially expressed miRNAs with overexpression and 3 miRNAs with underexpression in patients were evaluated through real-time PCR [17]. Xie et al. did the research on aberrant miRNAs used as biomarkers for the diagnosis of non-small-cell lung cancer (NSCLC). Their result indicated that mir-21 was detected with high expression in sputum specimen of patient [18]. Du Rieu et al. did the study on whether abnormal miRNA production for noninvasive precursor pancreatic intraepithelial neoplasia (PanIN) can be used as a potential early biomarker of pancreatic ductal adenocarcinoma (PDAC). They indicated that mir-21 was aberrantly expressed in early development of PanIN and it was worthy for further study as a biomarker for early detection of PDAC [19]. Habbe et al. did the research on the aberrant expressed miRNAs in intraductal papillary mucinous neoplasms (IPMNs). They showed that aberrant miRNA expression was an early event for pancreatic cancer, and miR-155 was worthy of further study as a biomarker for IPMNs in clinical samples [20].

Although several miRNA biomarkers have been reported in the above researches, only one or very few miRNAs are identified in each experiment which is involved in a single cancer type. The accuracy and specificity of some miRNAs are not so promising. In this paper, we analyze the miRNA expression patterns comparatively in cancer versus normal samples on early stage of eight prevalent cancer types. The datasets used in our paper are all RNA-Seq data from TCGA database to make a comprehensive identification of miRNA biomarkers for various cancers. We focus on identifying the specific miRNAs biomarkers including four aspects: (a) detecting differentially expressed miRNAs for each cancer type; (b) detecting specific differentially expressed miRNAs; (c) detecting specific miRNAs biomarkers; and (d) analyzing function and pathway of these miRNAs. Several identified miRNA biomarkers have been reported in existing researches and most of them could serve as potential candidate biomarkers for clinical early diagnosis.

## 2. Materials and Methods

### 2.1. miRNA Expression Datasets

The original miRNA expression data based on miRNA-Seq for eight prevalent cancer types are downloaded from the TCGA database [21]. The eight cancer types include prostate, thyroid, breast, head and neck, kidney, stomach, lung, and liver cancer. For each cancer type, we select the cancer samples which have corresponding samples from their normal tissues as the paired samples to identify cancer related miRNA biomarkers of different cancer types. Biomarkers can ensure that the prediction results could be well generalized to clinical research and utility by using these paired samples. Detailed information of the datasets used in this paper is listed in Table 1. In this paper, we only use the samples of cancer from pathologic stage I to detect miRNA biomarkers for cancers of early stage. The pathologic stage is collected from clinical patient information of TCGA database. The value of “reads per million miRNA mapped” is used as expression value of each miRNA.

### 2.2. Identification of Differentially Expressed miRNAs

For each cancer type, firstly, Wilcoxon signed-rank test is used to identify differentially expressed miRNAs between cancer samples and normal samples. Then, FDR controlling method is used to eliminate false discovery rate of the result by Wilcoxon signed-rank test. After the above processes, an improved fold change method is applied to identify the differential expressed miRNAs between cancer samples and normal samples.

In this paper, we use quantiles to calculate the fold change of each miRNA. A quantile is a cut point which divides a set of observational data into equal sized groups. So, the *q*-quantiles indicate the value that partitions a set of finite data into *q* groups of equal sizes. The number of *q*-quantiles of value is *q* − 1. For a variable *X*, the *k*th* q*-quantile (0 < *k* < *q*)  *Q*_*k*_ can be estimated using the following formula:

(1) $$\begin{array}{c} Q_{k}=x_{\left\lfloor h\right\rfloor }+\left(\;h-\left\lfloor \;h\;\right\rfloor \;\right)\left(\;x_{\left\lfloor h\right\rfloor +1}-x_{\left\lfloor h\right\rfloor }\;\right), \end{array}$$

where *h* = (*N* − 1)(*k*/*q*) + 1 and *N* is the sample size. The set of *q*-quantiles is defined as follows:

(2) $$\begin{array}{c} SQ\subseteq \left\{\;\left.\;Q_{1},\ldots ,Q_{k},\ldots ,Q_{q-1}\;\right\}\;\right.. \end{array}$$

Let *E* ∈ *ℜ*^*m*×*s*^ be the transcript measurements in a miRNA expression matrix with *m* miRNAs and *s* samples. Here, variable *X* is *E*[*i*, :]  (0 < *i* ≤ *m*) and the set of *q*-quantiles of miRNA *i* can be calculated using formulae (1) and (2). For miRNA *i* on normal samples and cancer samples, we can obtain two sets of* q*-quantiles for normal and cancer samples as *NQ*_*i*_ and *CQ*_*i*_, respectively. The fold change value of the miRNA *i* of *k*th* q*-quantile can be calculated using the following formula:

(3) $$\begin{array}{c} {\text{FC}}_{ik}=\left\{\begin{array}{cc} \frac{CQ_{ik}}{NQ_{ik}}-1 & \left(CQ_{ik}\geq NQ_{ik}\right)\\ 1-\frac{NQ_{ik}}{CQ_{ik}} & \left(CQ_{ik}<NQ_{ik}\right), \end{array}\right. \end{array}$$

where *CQ*_*ik*_ and *NQ*_*ik*_ are the expression values of miRNA *i* of *k*th* q*-quantile in cancer and normal samples, respectively. And then, the original fold change OFC_*i*_ of miRNA *i*, which is calculated by the average of FC_*ik*_ across all the samples, is defined as follows:

(4) $$\begin{array}{c} {\text{OFC}}_{i}=\frac{1}{q-1}\overset{q-1}{\underset{j=1}{\sum }}{\text{FC}}_{ik}, \end{array}$$

where *q* is the selected number of quantiles.

In this paper, the impact of standard deviation is also introduced to the original fold change. The improved fold change denoted by IFC_*i*_ of miRNA *i* is shown as follows:

(5) $$\begin{array}{c} {\text{IFC}}_{i}=\frac{{\text{OFC}}_{i}}{2\sqrt{\sigma \left(CQ_{i}\right)\sigma \left(NQ_{i}\right)}}, \end{array}$$

where *σ* is the standard deviation of sets of *q*-quantiles. In formula (5), not only the mean value of fold change but also the influence of variance across different samples is considered, which is a more effective and robust statistical analysis.

In this paper, *q* is set to 100 to calculate the quantiles. And then, we select differentially expressed miRNAs for each individual cancer type using the following rules: (1) the improved fold change (IFC) is less than −0.5 or greater than 0.5; and (2) *q*-value by FDR controlling for Wilcoxon signed-rank test is less than 0.05.

### 2.3. Identification of Specific Differentially Expressed miRNAs

The specific miRNA biomarkers for each cancer are selected, which can be used as discriminators for other cancers. We select specific differentially expressed miRNAs based on the differential expressed miRNA results from last section for each cancer. Let the improved fold change of miRNA *i* in cancer *C*_1_ be IFC_*i*_^*C*_1_^ and in other cancers *C*_2_, *C*_3_,…, *C*_*k*_  (*k* = 1, 2, …, 8) be IFC_*i*_^*C*_2_^, IFC_*i*_^*C*_3_^,…, IFC_*i*_^*C*_*k*_^. The miRNA *i* is regarded as a specific miRNA biomarker for cancer *C*_1_, if and only if miRNA *i* complied with the following formula:

(6) IFCiC1>t2,∑j=2kIFCiC1−IFCiCj×δiCj>t1×t2×k−1,

where *k* is the number of cancer types and *k* = 8 in this paper. *t*_1_ means the threshold of the ratio of different cancer types (the number of cancer types being considered); here we set *t*_1_ to be 0.75. *t*_2_ means the threshold of the improved fold change (IFC value); here we set *t*_2_ to be 0.5. *δ*_*i*_^*C*_*j*_^ is a factor to filter the miRNAs with low expression value and is defined as follows:

(7) δiCj=1,X−iC1≥X−iCj,δiCj=0,X−iC1<X−iCj,

where *X*_*i*_^*C*_*j*_^ is a vector which means the expression value of miRNA *i* on cancer *C*_*j*_ and ${\overset{-}{X}}_{i}^{C_{j}}$ is the average of *X*_*i*_^*C*_*j*_^.

For example, if miRNA *i* is upexpressed in cancer *C*_1_ (the first inequality in (6) is satisfied, which is |IFC_*i*_^*C*_1_^| > *t*_2_) and downexpressed or changed very little in other cancer types (the second inequality in (6) is satisfied, which is ∑_*j*=2_^*k*^(|IFC_*i*_^*C*_1_^ − IFC_*i*_^*C*_*j*_^|) × *δ*_*i*_^*C*_*j*_^ > *t*_1_ × *t*_2_ × (*k* − 1)), then this miRNA is a specific differentially expressed miRNA for cancer *C*_1_. It is a good discriminator for distinguishing cancer types between *C*_1_ and other *C*_*j*_  (*j* = 2,3,…, 8).

### 2.4. Identification of Specific miRNA Biomarkers

After obtaining the specific differentially expressed miRNAs for each single cancer type, we further select the circulating and upregulated miRNAs as biomarkers. The information of extracellular circulating miRNAs is downloaded from miRandola database [22]. Based on the source of extracellular miRNAs, the miRNAs in miRandola database are divided into four categories: Ago2, exosome, HDL, and circulating. In this paper, we only select the circulating miRNAs which are source of plasma and serum in miRandola database as candidate miRNA biomarkers.

For improving the sensitivity and specificity, we identify the combined miRNA biomarkers based on the specific single miRNA biomarkers. The rules of selecting combined biomarkers are considered here in order to identify *k*-miRNA discriminators. These *k*-miRNA discriminators are used as combined biomarkers for multiple cancer types, specific biomarkers for cancer types with similar survival rates (high survival rate, medium survival rate, and low survival rate), and a specific cancer type.

A computational process of finding the *k*-miRNA (*k* = 1,2, 3,4, 5) combination biomarkers is proposed in this paper to give a best distinction among the different cancer groups using a linear classifier [23]. Linear Discriminant Analysis (LDA) [23] is employed to evaluate the performance of *k*-miRNA combination biomarkers for multiple cancer types, cancers of similar survival rates, and a single cancer type. The overall accuracy is defined as the fraction of the total number of true positives and true negatives and the number of all the samples in the following:

(8) $$\begin{array}{c} \text{OA}=\frac{\left(\text{TP}+\text{TN}\right)}{N}, \end{array}$$

where TP is the number of true positives, TN is the number of true negatives, and *N* is the total number of samples.

The performance of all the *k*-miRNA combinations is evaluated using Leave-One-Out Cross Validation (LOOCV) method [24]. In each LOOCV step, one single sample is randomly chosen as the validation data, and the remaining samples are chosen as the training data to build the classifier using LDA. The OA value is calculated as the accuracy in this LOOCV step. This process is iterated until all of samples are selected as the validation data. Due to the computational complexity, the number of *k* combinations is set to be 1, 2, 3, 4, and 5 in this paper.

### 2.5. Function and Pathway Analysis

The functional annotation and pathway analysis are conducted using miEAA, a miRNA Enrichment Analysis and Annotation tool [25]. miEAA is a web-based system, which offers miRNA set enrichment analysis similar to Gene Set Enrichment Analysis (GSEA) [26]. The tool also provides rich functionality in terms of miRNA categories and contains over 14,000 miRNA sets, including pathways, diseases, organs, and target genes. In this paper, we perform the functional annotation and pathway analysis on specific upexpressed miRNAs for each cancer type among multiple cancer types, respectively.

## 3. Results and Discussion

The differentially expressed miRNAs in each cancer type are identified in order to study the specific roles of miRNAs involved in different cancer types. Due to the various alterations accumulated in the development of the oncogenesis, different cancers may have their specific miRNAs having differential expression. These miRNAs may be involved in some biological processes in the formation and progression of cancers. Similar to genes, several specific miRNAs are identified and used as targets of the prevention and diagnosis of cancers.

### 3.1. Differentially Expressed miRNAs

In this part, we use the strategy in the mentioned methods to identify the differentially expressed miRNAs in eight cancer types. We detect the upregulated and downregulated miRNAs, respectively, most of which may be involved in some important biology processes or pathways. The differentially expressed miRNAs of eight cancer types are summarized in Table 2. There are 79 differentially expressed miRNAs in breast cancer, among which 32 miRNAs are upregulated and 47 miRNAs are downregulated. There are 236 differentially expressed miRNAs in colorectal cancer, among which 82 miRNAs are upregulated and 154 miRNAs are downregulated. In the lung cancer results, 64 miRNAs are found to be differentially expressed, among which 46 miRNAs are upregulated and 18 miRNAs are downregulated. Interestingly, the number of differentially expressed miRNAs of different cancer types varies a lot. One reasonable explanation is that these miRNAs may reflect different regulatory mechanisms in different cancers and different cancers may accumulate different set of miRNAs.

Another interesting observation is that the number of upregulated and downregulated miRNAs in each cancer differs too much; that is, the ratio of upregulated and downregulated miRNAs differs a lot. In Table 2, we can see that there are more upregulated miRNAs than downregulated miRNAs in prostate and stomach cancers. Differently, the downregulated miRNAs in thyroid and liver cancers account for a major portion among the differently expressed miRNAs. It may possibly indicate the unique and similar characteristics of different cancer types.

### 3.2. Specific Differentially Expressed miRNAs

A detailed statistical analysis on specific differentially expressed miRNAs for single cancer type is conducted. The results are summarized in Table 3. There are 51 specific differentially expressed miRNAs in breast cancer, among which 21 miRNAs are upregulated and 30 miRNAs are downregulated. There are 180 specific differentially expressed miRNAs in colorectal cancer, among which 62 miRNAs are upregulated and 118 miRNAs are downregulated. In lung cancer, 46 miRNAs are found as specific differentially expressed miRNAs, among which 33 miRNAs are upregulated and 13 miRNAs are downregulated. Similarly, while the majority of differentially expressed circulating miRNAs in prostate and stomach cancers are upregulated, in kidney and liver cancers, the majority of such miRNAs are downregulated. The details of the specific differentially expressed miRNAs in eight cancers are illustrated in Table 3.

### 3.3. Specific miRNAs Biomarkers

The identification of specific miRNA biomarkers for each cancer is also performed in this part. For each cancer type, we use the strategy mentioned in Materials and Methods to find the miRNAs that have larger upregulation in each cancer type and have high expression value, and meanwhile the miRNAs have different changes or expression values in other cancer types. Such miRNAs can make a better distinction between one cancer and other cancer types.

**Figure 1:**
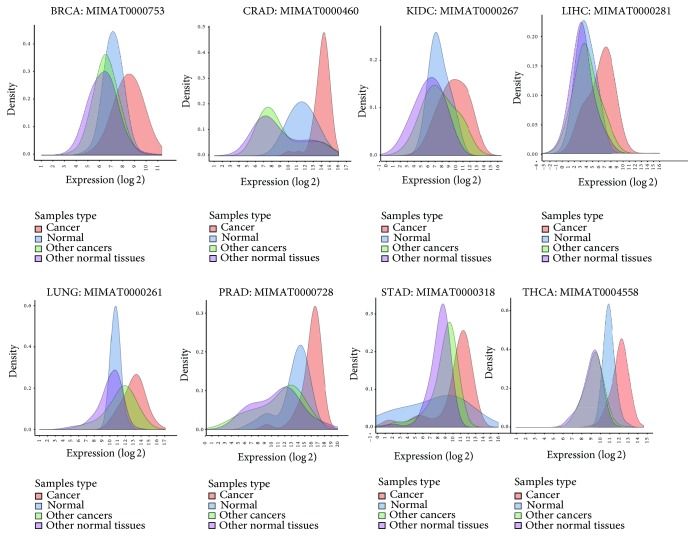
Specific differentially expressed miRNAs in eight cancer types.

The detailed information is illustrated in Figure 1. MiRNA MIMAT0000753 is taken as an example (the first one in Figure 1). It shows the expression distribution of miRNA MIMAT0000753 in breast cancer, normal breast, other cancers, and other normal tissues. MIMAT0000753 gets a fold change value of nearly 0.92 in breast cancer, which indicates its upregulation obviously. The approximate mean expression values are 470 and 160 in breast cancer and normal samples, respectively. The approximate mean expression values are 125 and 107 in other cancer and normal samples, respectively. So MIMAT0000753 gets a better performance in prostate cancer compared to other cancers. It could be used to distinguish prostate cancer from other cancer types.

In some cases, using one single gene or miRNA is not enough to distinguish the specific cancer. The identification of the combination of some miRNAs is an important step of differentially expressed miRNA analysis. In order to find the better combinations of miRNAs as biomarkers which can be used to distinguish the specific cancer, *k*-miRNA (*k* = 1,2, 3,4, 5) combinations of differentially expressed miRNAs are selected in this section. By identifying different miRNA combinations which have similar expression patterns in single cancer type but have different expression patterns in other cancers, we explore useful information of different and various mechanisms about carcinogenesis with single cancer type. In each *k*-miRNA combination, we calculate the classification capability in two aspects. One is the measurement of the *k*-miRNA in a single cancer dataset, which calculates the classification capability between cancer samples and the paired normal samples of the same cancer type. The other is the measurement of the *k*-miRNA in the datasets of multiple cancer types, which calculates the classification capability between one cancer type and the other cancer types using the fold change values of the cancer samples and the normal samples.  Table 4 gives the results of distinguishing ability of the top five-miRNA specific biomarker combinations in eight cancers, respectively.

In Table 4, accuracy 1 is the results obtained by classifier between cancer samples and their corresponding paired normal samples; and accuracy 2 is the results obtained by classifier between fold change values from each cancer type and fold change values from other cancer types. These miRNAs could be used as biomarkers for corresponding cancer, and simultaneously they are found to be effective discriminators for distinguishing each cancer from other cancer types. For example, in breast cancer the top five-miRNA of MIMAT(0000076 + 0000259 + 0000434 + 0000753 + 0003218) gets the accuracy of 77.02% in single dataset evaluation and accuracy of 79.54% in multiple cancer datasets evaluation process, which are the best results among other combinations. The mean value can reach 78.28%, which indicates that the five-miRNA combination could be good biomarkers for breast cancer.

As noted, several miRNAs in each combination have been reported to be related to different cancers. For BRCA, MIMAT0000076 (hsa-miR-21) is reported to be overexpressed in human breast cancer and associated with clinical stage, metastasis, and prognosis [27]. MIMAT0000259 (hsa-miR-182) and MIMAT0003218 (hsa-miR-92b) are found to be overexpressed in human breast tumor [28, 29]. MIMAT0000434 (hsa-miR-142) is reported to relate to the regulation of tumorigenicity of human breast cancer through the canonical WNT signaling pathway [30], and MIMAT0000753 (hsa-miR-342) regulates BRCA1 expression through modulation of ID4 in breast cancer [31].

For CRAD, MIMAT0000070 (hsa-miR-17) is found overexpressed in colon cancer and the expression is correlated with low survival rate in colorectal cancer patients [32], and MIMAT0000253 (hsa-miR-10a) is reported to be overexpressed in human colon cancer [33]. MIMAT0000067 (hsa-let-7g) is associated with tumorigenesis in colon cancer [34], and MIMAT0000438 (hsa-mir-152) is associated with cell proliferation, migration, and invasion in human cancer [35]. MIMAT0004598 (hsa-miR-141) is used as a novel biomarker for metastatic colon cancer [36].

For KIDC, MIMAT0000068 (hsa-miR-15a) is reported to be upregulated in urine of patients with renal cell carcinoma and undetectable in oncocytoma, other tumors, and urinary tract inflammation [37]. MIMAT0000076 (hsa-mir-21), MIMAT0004494 (hsa-miR-21-3p), and MIMAT0000267 (hsa-miR-210) are upregulated in renal cell carcinoma [37]. MIMAT0000255 (hsa-miR-34a) is reported as a specific oncogenic miRNA and shown to experience hypermethylation in kidney cancer cells [38].

For LIHC, MIMAT0000075 (hsa-miR-20a) is upregulated in liver cancer and correlated with hepatitis C virus-mediated liver disease progression [39]. MIMAT0000255 (hsa-miR-34a) and MIMAT 0002888 (hsa-miR-220) are reported as a potential therapeutic target in human cancer [40, 41]. MIMAT0000441 (hsa-miR-9) is upregulated in liver cancer tissues [42], and MIMAT0002871 (hsa-miR-500) is related to cancer survival [43].

For LUNG, MIMAT0000261 (hsa-miR-183) is reported to be overexpressed in lung cancer cell and suggested that high levels of CO2 increase these miRNA levels and it is related to mitochondrial oxygen consumption, ATP production, and cell proliferation [44]. MIMAT0000100 (hsa-miR-29b) is found to mediate NF-*κ*B signaling in KRAS-induced non-small-cell lung cancers (NSCLC) [45]. MIMAT0000432 (hsa-miR-141) and MIMAT0001080 (hsa-miR-196b) are upregulated in lung cancer [46]. MIMAT0004494 (hsa-miR-21-3p) is reported as biomarker to stratify for the subtype of lung cancer [47].

For PRAD, MIMAT0000074 (hsa-miR-19b) is reported to be related to prostate cancer and promoted prostate cell proliferation by targeting PTEN, PI3K/Akt pathway, and cyclin D1 [48]. MIMAT0000083 (hsa-mir-26b) is overexpressed in prostate cancer [49]. MIMAT0000432 (hsa-miR-141) is reported as a promising biomarker for prostate cancer [50]. MIMAT0000728 (hsa-miR-375) plays a dual role in prostate carcinogenesis [51], and the expression of MIMAT0001413 (hsa-miR-20b) is associated with progression of prostate cancer cells [52].

For STAD, MIMAT0000280 (hsa-miR-223) has significantly higher expression and plays a role in regulating cellular apoptosis, proliferation, and invasion in gastric cancer [53]. MIMAT0000071 (hsa-miR-17-3p) and MIMAT0000318 (hsa-miR-200b) are reported to be overexpressed in gastric cancer patients [54, 55]. MIMAT0000076 (hsa-miR-21) is used as potential diagnostic and prognostic biomarkers for gastric cancer [56], and MIMAT0004703 (hsa-miR-335-3p) is applied as prognostic signature in gastric cancer [57].

For THCA, MIMAT0000256 (hsa-miR-181a) is overexpressed in thyroid carcinoma and has been proposed to play a role in the pathogenesis, development, progression, metastasis, prognosis, and therapeutic response to chemo- and radiotherapy [58]. MIMAT0000257 (hsa-miR-181b) is reported as a key regulator of the oncogenic process in cancer [59]. MIMAT0000278 (hsa-miR-221), MIMAT0000279 (hsa-miR-222), and MIMAT0004558 (hsa-miR-181a-2-3p) are upregulated in thyroid carcinoma [58].

### 3.4. Function and Pathway Analysis

In this section, the function annotation and pathway enrichment are performed using miEAA database. Firstly, we analyze the enriched pathways on specific upexpressed miRNAs for each cancer type among multiple cancer types, respectively. Then, we select the enriched pathways in most cancer types and sort them by the hint ratio.  Table 5 gives top enriched pathway information with top ranks according to their hint ratio by miEAA [25]. The category means the source of pathway databases, the term means the enriched pathways, and the hint ratio means the enrichment score of miRNAs. From Table 5, we can see that the top enriched pathways are derived from Kyoto Encyclopedia of Genes and Genomes (KEGG) [60] and Wiki Pathways [61].

The first enriched pathway is apoptosis, which is described by its morphological characteristics and contributed to the high rate of cell loss in malignant tumors [62]. There are two enriched pathways, miRNAs involved in DDR and DNA damage response, which are related to DNA damage. There is an incontrovertible link between DNA damage and neoplastic phenotype in cancer [63]. Another interesting pathway is endocytosis, which entails selective packaging of cell-surface proteins and can be modified in cancer [64]. There are six signaling pathways in the enrichment results. Cellular signaling pathways are interconnected to form complex signaling networks and altered in cancer cells representing a major intellectual challenge [65].

## 4. Conclusions

Early detection of cancer is a very important and necessary way for cancer prevention. The biomarkers, as effective indicators to distinguish between cancer and normal samples or among different groups of cancer samples, are more and more used in cancer mechanism studies and clinical detection. The research of miRNA biomarkers is attracting more attention. But some of the biomarkers lack specificity and there are no effective common biomarkers for multiple cancer types. In this paper, we mainly focus on the identification of specific miRNAs and the common miRNA biomarkers. The miRNA expression data from RNA-Seq for eight cancer types are obtained from TCGA database, and the circulating miRNA information is collected from miRandola database.

For each cancer type, we apply Wilcoxon signed-rank test, which eliminates false discovery rate by using FDR controlling method, and improved fold change (FC) to identify miRNAs which have differential expressions between cancer samples and their corresponding normal samples. Then, the specific miRNA biomarkers for each cancer are further selected to act as discriminators for other cancers. After obtaining the specific differentially expressed miRNAs for each single cancer type, we further select the circulating and upregulated miRNAs as biomarkers. Several identified miRNA biomarkers in this paper are matched well with those reported in existing literatures and most of them could be taken as potential candidate indicators for clinical early diagnosis applications.

## Figures and Tables

**Table 1:** The detailed information of cancer miRNA expression datasets.

Cancer type	Abbreviation	Number of normal samples	Number of cancer samples
Breast	BRCA	87	157
Colorectal	CRAD^1^	22	96
Kidney	KIDC^2^	105	350
Liver	LIHC	50	192
Lung	LUNG^3^	91	459
Prostate	PRAD	52	149
Stomach	STAD	45	62
Thyroid	THCA	59	325
All		511	1790

^1^CRAD contains COAD (colon adenocarcinoma) and READ (rectum adenocarcinoma).

^2^KIDC contains KIRC (kidney renal clear cell carcinoma) and KIRP (kidney renal papillary cell carcinoma).

^3^LUNG contains LUSC (lung squamous cell carcinoma) and LUAD (lung adenocarcinoma).

**Table 2:** The differentially expressed miRNA in eight cancer types.

Cancer types	Upregulation	Downregulation	All	Ratio of up/down
BRCA	32	47	79	0.681
CRAD	82	154	236	0.533
KIDC	33	59	92	0.559
LIHC	33	67	100	0.493
LUNG	46	18	64	2.556
PRAD	71	3	74	23.667
STAD	77	14	91	5.500
THCA	19	48	67	0.396

**Table 3:** The specific differentially expressed miRNAs in eight cancer types.

Cancer types	Upregulation	Downregulation	All	Ratio of up/down
BRCA	21	30	51	0.700
CRAD	62	118	180	0.525
KIDC	11	24	35	0.458
LIHC	19	41	60	0.463
LUNG	33	13	46	2.538
PRAD	19	2	21	9.500
STAD	51	8	59	6.375
THCA	15	25	40	0.600

**Table 4:** Five-miRNA biomarker for eight cancers.

Cancer types	Markers	Accuracy 1	Accuracy 2	Mean
BRCA	MIMAT(0000076 + 0000259 + 0000434 + 0000753 + 0003218)	0.7702	0.7954	0.7828
CRAD	MIMAT(0000067 + 0000070 + 0000253 + 0000438 + 0004598)	0.8300	0.8657	0.8478
KIDC	MIMAT(0000068 + 0000076 + 0000255 + 0000267 + 0004494)	0.7473	0.8000	0.7736
LIHC	MIMAT(0000075 + 0000255 + 0000441 + 0002871 + 0002888)	0.7021	0.7726	0.7373
LUNG	MIMAT(0000100 + 0000261 + 0000432 + 0001080 + 0004494)	0.7311	0.7815	0.7563
PRAD	MIMAT(0000074 + 0000083 + 0000432 + 0000728 + 0001413)	0.8060	0.8366	0.8213
STAD	MIMAT(0000071 + 0000076 + 0000280 + 0000318 + 0004703)	0.7853	0.8029	0.7941
THCA	MIMAT(0000256 + 0000257 + 0000278 + 0000279 + 0004558)	0.6876	0.8805	0.7841

**Table 5:** Enriched pathway information of eight cancer types.

Category	Term	Hint ratio
KEGG	Apoptosis (hsa04210)	1.000
Wiki Pathways	miRNAs involved in DDR (WP1545)	1.000
Wiki Pathways	Leptin signaling pathway (WP2034)	1.000
KEGG	Adipocytokine signaling pathway (hsa04920)	0.875
Wiki Pathways	Estrogen signaling pathway (WP712)	0.875
Wiki Pathways	Insulin signaling (WP481)	0.875
Wiki Pathways	DNA damage response (WP707)	0.875
Wiki Pathways	IL 4 signaling pathway (WP395)	0.875
KEGG	Wnt signaling pathway (hsa04310)	0.875
KEGG	Endocytosis (hsa04144)	0.875
